# Episodic Disturbance from Boat Anchoring Is a Major Contributor to, but Does Not Alter the Trajectory of, Long-Term Coral Reef Decline

**DOI:** 10.1371/journal.pone.0144498

**Published:** 2015-12-30

**Authors:** Graham E. Forrester, Rebecca L. Flynn, Linda M. Forrester, Lianna L. Jarecki

**Affiliations:** 1 Department of Natural Resources Science, University of Rhode Island, 1 Greenhouse Road, Kingston, Rhode Island, 02881, United States of America; 2 Department of Biological Sciences, University of Rhode Island, 120 Flagg Road, Kingston, Rhode Island, 02881, United States of America; Leibniz Center for Tropical Marine Ecology, GERMANY

## Abstract

Isolating the relative effects of episodic disturbances and chronic stressors on long-term community change is challenging. We assessed the impact of an episodic disturbance associated with human visitation (boat anchoring) relative to other drivers of long-term change on coral reefs. A one-time anchoring event at Crab Cove, British Virgin Islands, in 2004 caused rapid losses of coral and reef structural complexity that were equal to the cumulative decline over 23 years observed at an adjacent site. The abundance of small site-attached reef fishes dropped by approximately one quarter after the anchoring event, but this drop was not immediate and only fully apparent two years after the anchoring event. There was no obvious recovery from the impact, and no evidence that this episodic impact accelerated or retarded subsequent declines from other causes. This apparent lack of synergism between the effect of this episodic human impact and other chronic stressors is consistent with the few other long-term studies of episodic impacts, and suggests that action to mitigate anchor damage should yield predictable benefits.

## Introduction

Many ecological communities have exhibited progressive shifts in composition over the past half century. Declines in terrestrial, marine, and freshwater ecosystems are well documented, but isolating the relative influence of the various factors that cause these declines remains a challenging problem [1, 2]. One major difficulty is separating the potential causes of decline from their effects [3, 4]. Coral reef habitats have the highest biodiversity of any marine habitat, and perform key ecosystem services for many coastal communities [5], but corals are declining globally [6, 7], and reefs are losing three-dimensional complexity [8]. Both diminishing coral cover and reef complexity negatively impact reef fishes, many of which utilize the reef structure as refuge [9, 10]. As with declines in most habitats, reef degradation appears to be caused by the integrative effects of natural disturbances (e.g. hurricanes) and anthropogenic stressors [11–13]. Anthropogenic stressors are mix of local and global factors [14], including overfishing, coastal pollution and development, tourism, climate change and introduced species [12, 15–19]. Disentangling the combined effect of multiple stressors is challenging. In the simplest case they may act independently so that their combined effects are additive. Alternatively, they may interact either synergistically, whereby their combined effect is greater than the sum of their isolated effects, or antagonistically, in which case their combined effect is less than the sum of the individual effects [20–22]. In this study, we isolate the cause of an episodic human impact and quantify its long-term impact on a coral reef community relative to the combined effect of all other stressors [23, 24].

Impacts from overfishing [25, 26], and more recently from climate change [27], have often been viewed as the major drivers of reef decline. More recently, an alternate hypothesis proposes that a suite of local drivers collectively associated with coastal development, pollution, and tourism have been understudied and perhaps underestimated [19]. Some support for this hypothesis is provided by spatial surveys that show negative correlations between human population density and reef community composition across Pacific Islands [28] and Caribbean sites [18]. Although their impact is not easily separated from the overall human footprint on coral reefs, recreational visitors often form a major fraction of the total human population around coral reefs and visitor density is also negatively correlated with coral abundance across the Caribbean [19]. We focused on one consequence of increased visitation by tourists, anchor damage from recreational boats. As a consequence of a rise in tourism, recreational boat traffic is increasing rapidly in many areas [29, 30]. Boat anchoring has long been acknowledged as a source of damage to coral reefs [31], but, compared to other human impacts, its effects have received virtually no formal study [32].

**Fig 1:**
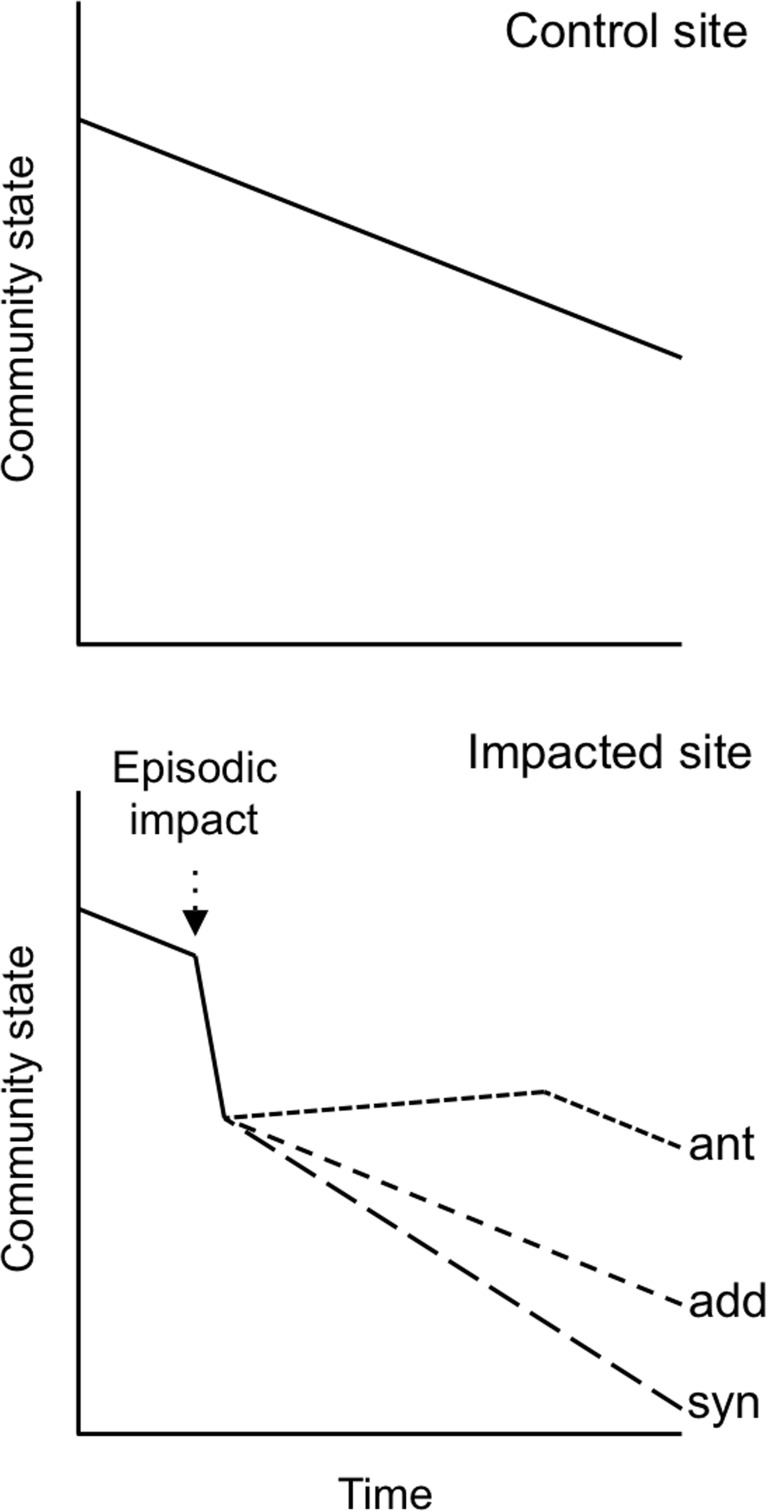
Three potential trajectories of community change after an episodic disturbance (Impacted site), against a backdrop of gradual decline due in response to chronic stressors (Control site) [24]. An antagonistic effect of the episodic impact is indicated by recovery before eventual resumption of the pre-impact decline (ant), an additive effect is indicated by an almost immediate continuation of decline at the pre-impact rate (add), and synergism is revealed by resumption of community decline but at an accelerated rate (syn).

In this study, our objectives were (i) to test the effect of a single anchoring event on a coral reef community and (ii) assess its contribution to long-term change at the site. This opportunity arose unexpectedly when a large (50 m) vessel anchored overnight on a reef in the British Virgin Islands (BVI) in 2004 and damaged part of it. Because the reef has been surveyed annually from 1992 to the present, we could isolate the impact of the anchoring event by testing for a divergence between the impacted and unimpacted parts of the site that coincided with the event (using a Before-After-Control-Impact [BACI] design [33]). Of greatest conceptual interest was whether the impact from the anchoring event altered the subsequent trajectory of change at the site, by either magnifying or diminishing the subsequent effects of other stressors (Fig 1, see also [24]). We focused our analysis on three important aspects of the coral reef community state: (1) the abundance of scleractinian corals—the key foundation species [34] in this ecosystem, (2) reef structural complexity–an index of the quality of habitat for many fishes and invertebrates [8], and (3) the density of fishes–important consumers on reefs and the main source of reef-derived food for people.

## Methods

### Study Area

Tourism is the second largest contributor to the BVI economy (27% of gross domestic product in 2013), and the territory hosted over 355,000 overnight visitors in 2013 [35]. Most tourism income in the BVI is generated by the yacht chartering industry [36]. Currently, there are 1100–1500 charter yachts (12–16 m length), plus a growing number of “mega-yachts” (>45 m in length), operating in the territory’s 150 km^2^ of coastal waters (Janet Oliver, BVI Charter Yacht Society, personal communication). In an effort to reduce the need for anchoring, > 200 yacht moorings have been installed throughout the BVI (Nancy Pascoe, National Parks Trust of the Virgin Islands, personal communication).

### Long-term coral reef monitoring

**Fig 2:**
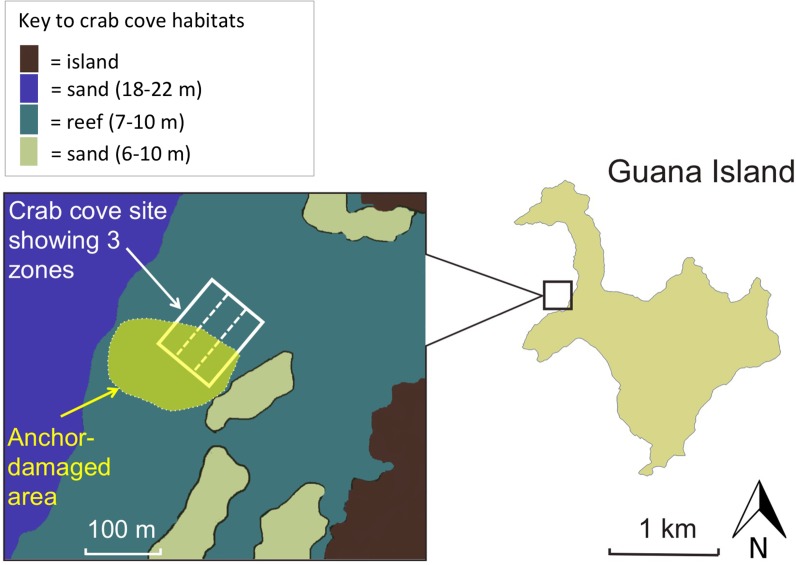
A map of the Crab Cove study site near Guana Island, British Virgin Islands. The image of Crab Cove shows the portion of the site damaged by the one-off anchoring event in 2004, and the three zones within the site that are used to account for the offshore gradient in reef structure.

Our analysis isolated the impact of a single severe anchoring event at Crab Cove, near Guana Island, BVI (Fig 2), an approximately 1 ha site that has been monitored annually from 1992 to the present [37]. All reef monitoring was done on SCUBA. Each year, four to eight 30 m transects were placed at haphazard locations within the site. Fish were counted using 30 x 1.5 m belt transects, using a T-shaped bar to delineate the transect width. We counted all small-to-medium sized diurnal species that were relatively site-attached, excluding some cryptic benthic gobies and blennies that are hard to census visually. Ninety one fish species were counted, but the 17 most common species made up 60% of the total number of fish counted (S1 Table). To estimate the cover (%) of scleractinian corals, we used the linear point-intercept method, in which a diver swam along the transect and identified the material under the transect at 0.25 m intervals (n = 120 points per transect) [38]. To estimate reef structural complexity, we used a variant of the consecutive height difference method, which has been shown to perform effectively in field comparisons with other methods [39]. The transect tape was stretched tight across the reef surface, and we measured the distance in cm perpendicular from the tape to the reef surface every meter for the first 10 m (*n* = 10 height measurements per transect). Structural complexity was calculated as the square root of the sum of the squared differences between successive height measurements [39].

### Ethics statement for field studies

This research was conducted with the approval of the BVI Department of Conservation and Fisheries, and fish counts were approved by the URI Institutional Animal Care and Use Committee (protocol AN13-04-016).

### Documenting a single anchoring event by a 50 m vessel

On 7 July 2004, a 50 m vessel, the Holo-Kai, anchored overnight on part of the site (S1 Fig). On the following day, the reef was assessed and mapped by divers. It appeared that heavy chains from the three anchors deployed had damaged roughly 1.5 ha of reef, including about half of the monitoring site (Fig 2). This survey of the area revealed symptoms of apparent recent anchor damage: newly overturned, broken, and scarred coral colonies, plus recently bent and broken soft corals (S2 Fig). To assess the extent of damage symptoms, and check that the symptoms reflected anchor damage, rather than other unknown impacts, we placed 30 m transects at haphazardly selected locations inside (n = 6) and outside (n = 6) the damaged area. Divers used the linear point-intercept method to estimate the cover (%) of the aforementioned damage symptoms. Surveys were conducted at Crab Cove in late July and October 2004. In October, we surveyed an additional five reef sites around the perimeter of Guana Island (*n* = 3 transects per site) as a broader check of the site-specificity of damage symptoms.

### Analysis of the anchoring impact

The timing and location of the anchoring event created the unexpected opportunity to assess its effect as a BACI design, although this approach is more commonly used to analyze planned environmental impacts [33]. We mapped transect locations each year, so comparing the maps of transect location to the map of the damaged area allowed us to retrospectively classify all transects as lying outside (control) or inside (impact) the anchor-damaged area. There was also a visually obvious spatial gradient of increasing coral cover with increasing distance from shore throughout the study (S2 Table), so we divided the site into three zones (Fig 2) and used the maps of transect placement to retrospectively classify each transect by zone. Because transect placement was made independent of anchor-damage and zone, the number of transects per zone in the control and impact areas varied each year and was zero in some years.

Various statistical models have been used to analyze BACI designs, and we used two of these models to ensure that the results were not influenced by the specifics of any given model (analyses were performed using SPSS v. 22, IBM Corporation, and SAS v. 9.3, SAS Systems Inc.). The first method was one of the simplest BACI approaches [33]. For each date when transects were sampled in the control and impact parts of the site, and both transects were also in the same zone, we calculated the difference between control and impact measurements. The before sample of differences was then compared to the after sample using a *t*-test [33]. The second method used to isolate the effect of the anchoring event was a linear mixed model, which accounts for more sources of variation in the data. Zones within the site (1, 2 or 3) were treated as spatial replicates (i.e. subjects), to account for the spatial gradient in coral cover. For this analysis, a replicate is thus the mean of the transects within a given zone in a given year (the number of transects from which each mean was derived is shown in S3 Table). A fixed categorical factor accounted for anchor damage (whether transects were inside or outside the anchor-damaged part of the site). There were two repeated factors: (i) year of sampling and (ii) before vs. after the anchoring event (i.e. years were classified into two groups—those before and those after the anchor damage). We checked data for normality, and examined different temporal covariance structures and the most appropriate was the first order autoregressive covariance structure (AR1), which has homogeneous variances and correlations that decline across years exponentially. Our main interest was the interaction between the “before vs. after” and “control vs. impact” effects, which tests the impact of the 2004 anchoring event [33].

We used both models to estimate the magnitude of the anchoring impact (Tables 1 and 2). From the linear mixed model, the marginal mean within the damaged area before the anchoring event (*B*_*in*_) minus the mean after (*A*_*in*_) is a crude but simple estimate of the loss from the anchoring event (Table 1). With the gradual decline (year effect) accounted for, the difference between the before (*B*_*out*_) and after (*A*_*out*_) means in the control area measures the background level of change. To estimate the anchoring effect we subtracted the change in the control area (*B*_*out*_—*A*_*out*_) from that in the damaged area (*B*_*in*_ *—A*_*in*_) (Table 1). From the *t*-test, we estimated the size of the anchoring effect by subtracting the mean of control-impact differences before the anchoring event from the mean difference after the event (Table 2).

**Table 1:** Three measures of community state (coral cover, reef structural complexity, and fish density) before and after the 2004 anchoring event, in the damaged and undamaged part of the site. Shown are marginal means (±SE) from the linear mixed model, which are adjusted for other variables in the model. Calculation of the anchoring effect is explained in the text.

a) Absolute coral cover (%)								
		Before		After		Change (After—Before)		Anchoring effect
	Damaged	24.1	(±3.8)	12.0	(±4.0)	-12.1		-11.5
	Undamaged	21.5	(±4.1)	20.9	(±4.0)	-0.6		
b) Index of reef structural complexity (cm)								
		Before		After		Change (After—Before)		Anchoring effect
	Damaged	37.8	(±6.3)	26.6	(±6.2)	-11.3		-8.7
	Undamaged	37.1	(±6.4)	34.5	(±6.3)	-2.6		
c) Fish density (# per 45m^2^)								
		Before		After		Change (After—Before)		Anchoring effect
	Damaged	60.4	(±6.0)	51.2	(±5.9)	-9.2		-18.0
	Undamaged	64.8	(±6.1)	73.6	(±6.3)	+8.9		

**Table 2:** Control-impact differences before and after the anchoring event. The raw data for this comparison were simultaneous measurements in the anchor damaged part of the site (impact) and the undamaged area (control). Displayed are means (±SE) of the difference between the paired measurements. Calculation of the anchoring effect is explained in the text.

Control–impact difference	Before		After		Anchoring effect
Absolute coral cover (%)	4.3	(±1.4)	-7.3	(±1.2)	-11.6
Reef structural complexity (cm)	0.4	(±1.6)	-6.8	(±1.6)	-7.2
Fish density (# per 45m^2^)	-3.2	(±5.3)	-17.4	(±5.7)	-14.2

## Results

### Damage symptoms

Just after the anchoring event in late July 2004, symptoms of recent coral damage (S3 Fig) were ten times more common in the affected part of Crab Cove than in the unaffected part (S3 Fig). This difference was still apparent in October, at which time symptoms were also far more common in the impacted part of Crab Cove than in five other nearby sites (S4 Fig). The fact that damage symptoms were effectively restricted to the area where anchoring occurred strongly suggests that the anchoring event caused the coral damage.

### Coral cover

**Fig 3:**
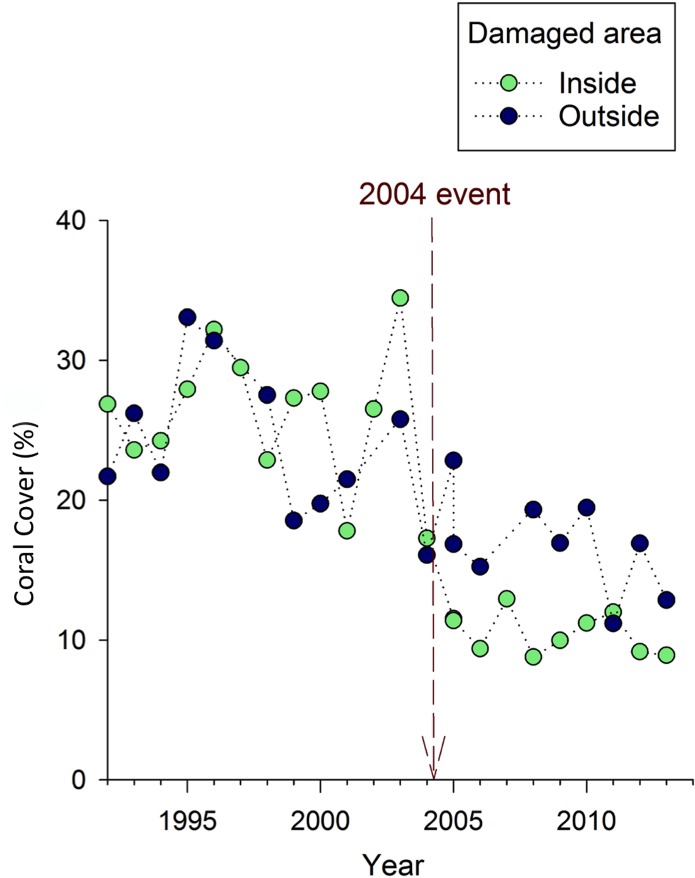
Mean absolute coral cover (%) over time at Crab Cove, showing changes inside and outside the area damaged by the 2004 anchoring event. The dotted vertical line indicates the timing of the 2004 anchoring event.

Within the area damaged by the Holo-Kai, there was an abrupt loss of coral that occurred directly after the anchoring event, which did not occur outside of the area affected by the Holo-Kai (Fig 3). In both parts of the site, there was also a gradual decline in coral cover from 1992-present (Fig 3). A significant impact of the anchoring event was supported by the results of both the BACI analysis and the linear mixed model. Support from the linear mixed model was provided by a significant interaction between the “control versus impact” and “before versus after” effects (*F*_1,64_ = 63.4, *p* = 0.012). The *t*-test supported an anchoring impact because the control-impact differences were greater after the anchoring event than before (*t*_14_ = 5.98, *p* = 0.0004).

Both statistical models also produce similar estimates of the magnitude of the anchoring impact—a drop in absolute coral cover of 11–12% (Tables 1 and 2). To put the anchoring impact in context, from 1992-present absolute coral cover declined from approximately 33% to 8% in the anchor-damaged area (Fig 3), which suggests that almost half of the overall long-term decline was attributable to the one-time anchoring event in 2004.

### Topographic relief

**Fig 4:**
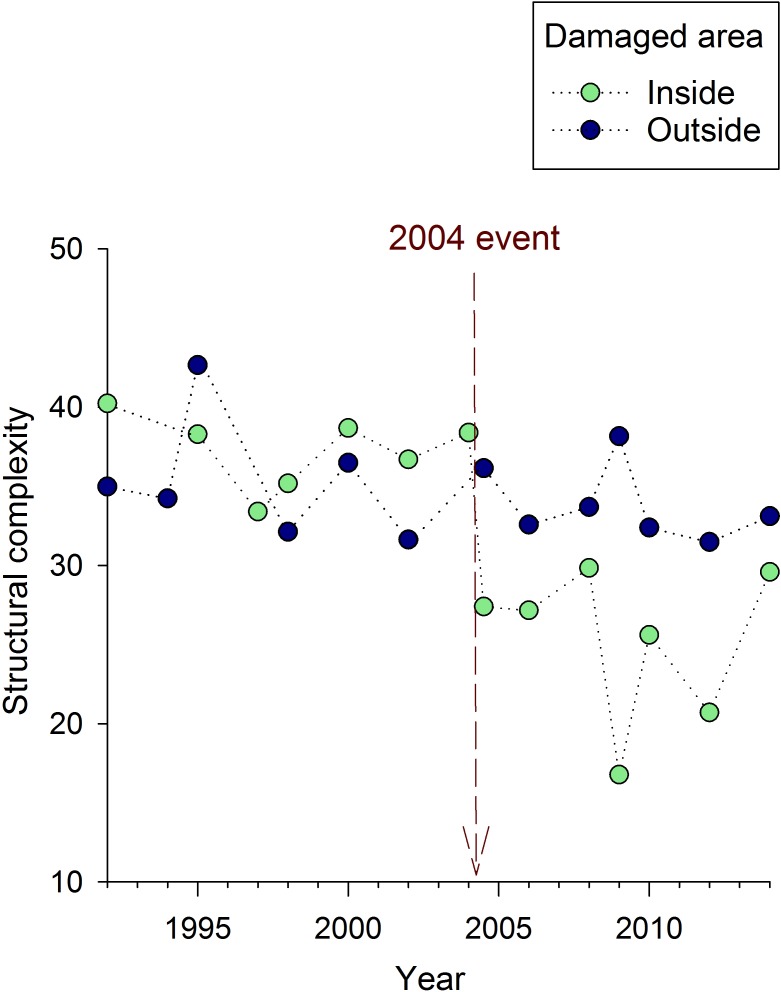
Mean reef structural complexity over time at Crab Cove, showing changes inside and outside the area damaged by the 2004 anchoring event. The dotted vertical line indicates the timing of the 2004 anchoring event.

The qualitative pattern of change in reef structural complexity was very similar to that described for coral cover. In the area damaged by the Holo-Kai, there was a rapid drop in structural complexity that was not observed outside of the anchor-damaged area (Fig 4). A significant impact of the anchoring event was supported by the results of both the *t*-test (*t*_9_ = 3.85, *p* = 0.006) and the linear mixed model (interaction between “inside versus outside” and “before versus after” effects; *F*_1,33_ = 10.8, *p* = 0.002). Throughout the site, there also appeared to be a gradual decline in structural complexity from 1992-present (Fig 4).

The linear mixed model and *t*-test yielded similar estimates of the magnitude of the anchoring impact; a drop in the mean structural complexity index of 7.2 and 8.6 cm respectively (Tables 1 and 2). Within the damaged part of the site, mean structural complexity dropped by 16.6 cm over the course of the study (1992 mean = 40.2 cm and 2014 mean = 23.6 cm; Fig 4). Comparing these estimates suggests that close to half (≈7.2–8.6 cm) of the long-term decline in topographic relief (≈16.6 cm) was attributable to the anchoring event in 2004.

### Fish density

**Fig 5:**
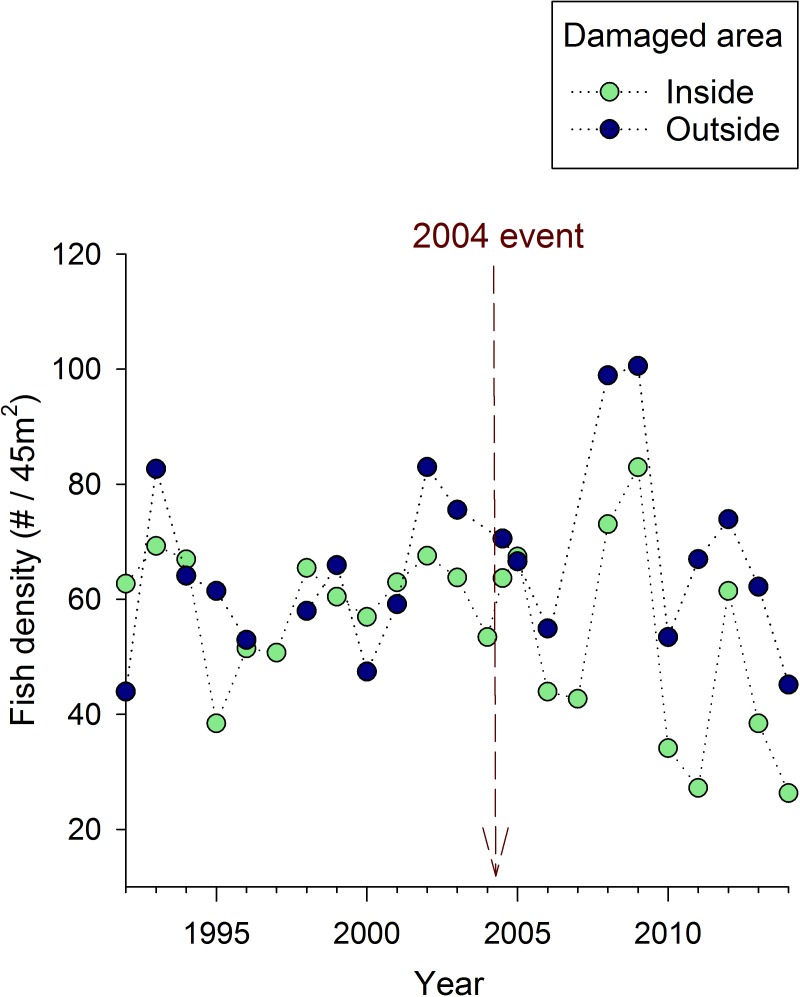
Mean adult fish density over time at Crab Cove, showing changes inside and outside the area damaged by the 2004 anchoring event. The dotted vertical line indicates the timing of the 2004 anchoring event.

Fish densities also appear to be reduced by the anchoring event, but the effect was less pronounced than the effect on corals and topographic relief (Fig 5). Both the *t*-test (*t*_13_ = 3.27, *p* = 0.006) and the linear mixed model (interaction between “inside versus outside” and “before versus after” effects; *F*_1,58_ = 5.03, *p* = 0.03) suggested a significant impact of the anchoring event. Inspection of the temporal trends suggests that, unlike the steady long-term decline in coral cover and topographic relief, fish densities appeared to fluctuate around a relatively constant long-term average (Fig 5). Nonetheless, from 2006 onward, densities were generally lower in the anchor-damaged part of the site than the unimpacted area (Fig 5). The lack of a distinct break-point immediately after the anchoring event reduces the accuracy of the before-after comparison as an estimate of the anchoring impact. With this caveat in mind, using the same calculations described for coral and topographic relief suggests that fish density dropped by a quarter after the anchoring event (down by ≈14–18 fish per 45 m^2^ from the pre-anchoring mean of 63 fish per 45 m^2^).

## Discussion

This study provided a rare opportunity to make a clear causal connection between an episodic human impact, subsequent damage symptoms and the long-term community impact. Pinpointing the contribution of different factors to widespread reef declines is challenging [1, 2], in part because it is difficult to track and quantify the potential causes of decline independent from their effects [4]. By isolating the impact of the Holo Kai anchoring event, we established that the amount of coral mortality inflicted in one night by a large vessel approximately equaled the cumulative loss over 23 years caused by all other factors combined. The drop in absolute coral cover attributed to the Holo-Kai in one night (12%) was also substantial relative to the total change in coral cover over 23 years at seven other BVI sites, where the mean absolute cover declined from 30% in 1992 to 18% in 2013 [37]. Moreover, the Holo-Kai impact was also substantial relative to the most recent Caribbean-wide estimate of long-term coral decline, a reduction in absolute cover of 19% in 40 years [19].

Our results also indicate a strong direct effect of the anchoring event on reef structure, and an indirect effect on fish populations. The proportional impacts on reef structural complexity and fish density attributable to the anchoring event are of substantial magnitude relative to long-term region-wide declines in these variables [8, 40, 41]. The relative timing of the impacts, the rapid coincident loss of coral and structural complexity followed two years later by a reduction in fish density is also consistent with the relative timing of region-wide declines of these variables [40, 41]. The causal connection between them–corals create the structural complexity that provides shelter for fish–is well-established by experimental habitat manipulations [42–46] and cross-site comparisons [47–50].

Our most significant finding was that there was no apparent recovery from the anchoring impacts, and no evidence that the impacts accelerated or retarded subsequent declines from other causes. Factorial experiments provide the simplest and most reliable tests for interactive effects of environmental stressors [20] and recent reviews of experimental studies conclude that additive, antagonistic and synergistic effects are relatively equal in likelihood [21, 22, 51–53]. Virtually all studies reviewed, however, address short-term behavioral, physiological or demographic responses. To inform conservation actions, it is critical to determine whether similar generalizations apply to long-term community change. Our long-term analysis revealed no evidence of synergism or antagonism, which would be indicated in the BACI context by an obvious post-anchoring change in trajectory that occurred in the impact area but not the control area, assuming that other unidentified stressors affected the control and impact area equally (Fig 1 and see also [24]). Using a different approach to ours, the long-term impact on coral cover of another episodic human impact (bleaching attributed to climate change) was also shown to be additive or weakly antagonistic when assessed against that of a chronic stressor (fishing impacts inferred from protection in reserves) [54]. We know of no other controlled assessments of long-term anchoring impacts on coral communities, but the immediate symptoms (scarred, broken and overturned corals) are superficially similar to those produced by hurricanes [23, 24]. A meta-analysis of post-1980 hurricane impacts in the Caribbean revealed a pattern of additive impacts, in which coral cover declined by almost one fifth on average in the year after a storm, but thereafter simply continued to steadily decline at the pre-storm rate [24]. This pattern of impacts is remarkably similar to the anchoring impact we detected, and suggests the general hypothesis that episodic disturbances tend to have additive long-term effects.

The apparent lack of synergism between the long-term effect of this episodic human impact and other chronic stressors is important for conservation as human activity shifts the natural disturbance regime in ecosystems, adds new disturbances, and transforms previously irregular events into more common and persistent stresses [55]. A companion study, based on a synoptic survey of 25 reefs in 2014, showed that anchor damage is now spatially widespread in the BVI and that coral cover, structural complexity, and fish density are reduced by half at regularly anchored sites [56]. Actions to manage local stressors like boat anchoring are thus important in their own right, but they are also increasingly motivated by the goal of compensating for the effects of global stressors such as climate change [14, 57, 58]. This approach assumes that stressors interact additively or synergistically, so that their combined effect is equal to, or greater than, the sum of their isolated effects [54]. Additive effects can be quantified and prioritized for action relatively simply [59], and our results suggest that action to mitigate anchor damage should yield predictable benefits. Synergisms and antagonisms are, in contrast, more likely to yield ecological “surprises” [60–62]. Dealing with antagonistic interactions is problematic conceptually, but local stressors that have synergistic interactions are potential priorities for management because they magnify the influence of other stressors, and so minimizing their effects may have the greatest net benefit in the long-term [60]. A priority for future research is understanding the ecological mechanisms that dictate whether and how the effects of stressors interact to create differing trajectories of long-term change [4].

## Supporting Information

S1 Fig The Holo Kai at anchor in Crab Cove on 07 July 2004. (TIF)

S2 Fig Recently overturned and scarred corals considered symptoms of damage from the Holo Kai’s anchors and anchor chain. Photos taken in late July 2004.
(TIF)

S3 Fig Percent cover of recent coral-damage symptoms at Crab Cove a few days after the anchoring event (July) and 3 months later (October). (TIF)

S4 Fig Percent cover of recent coral-damage symptoms 3 months after the anchoring event. Damage symptoms in Crab Cove are shown alongside symptoms at 5 other sites around Guana Island.
(TIF)

S1 Table A list of the 17 most common fish species surveyed. Sixty percent of fish counted belonged to these 17 species.
(PDF)

S2 Table Time-averaged mean coral cover in the three zones within the Crab Cove site. (PDF)

S3 Table Sample sizes for the linear mixed model. The number of transects sampled within each zone in each year. (PDF)
