# Supplementary figures and images for: Episodic Disturbance from Boat Anchoring Is a Major Contributor to, but Does Not Alter the Trajectory of, Long-Term Coral Reef Decline
Source: PLoS One. 2015 Dec 30;10(12):e0144498. doi: 10.1371/journal.pone.0144498 (PMC4696730; doi:10.1371/journal.pone.0144498)

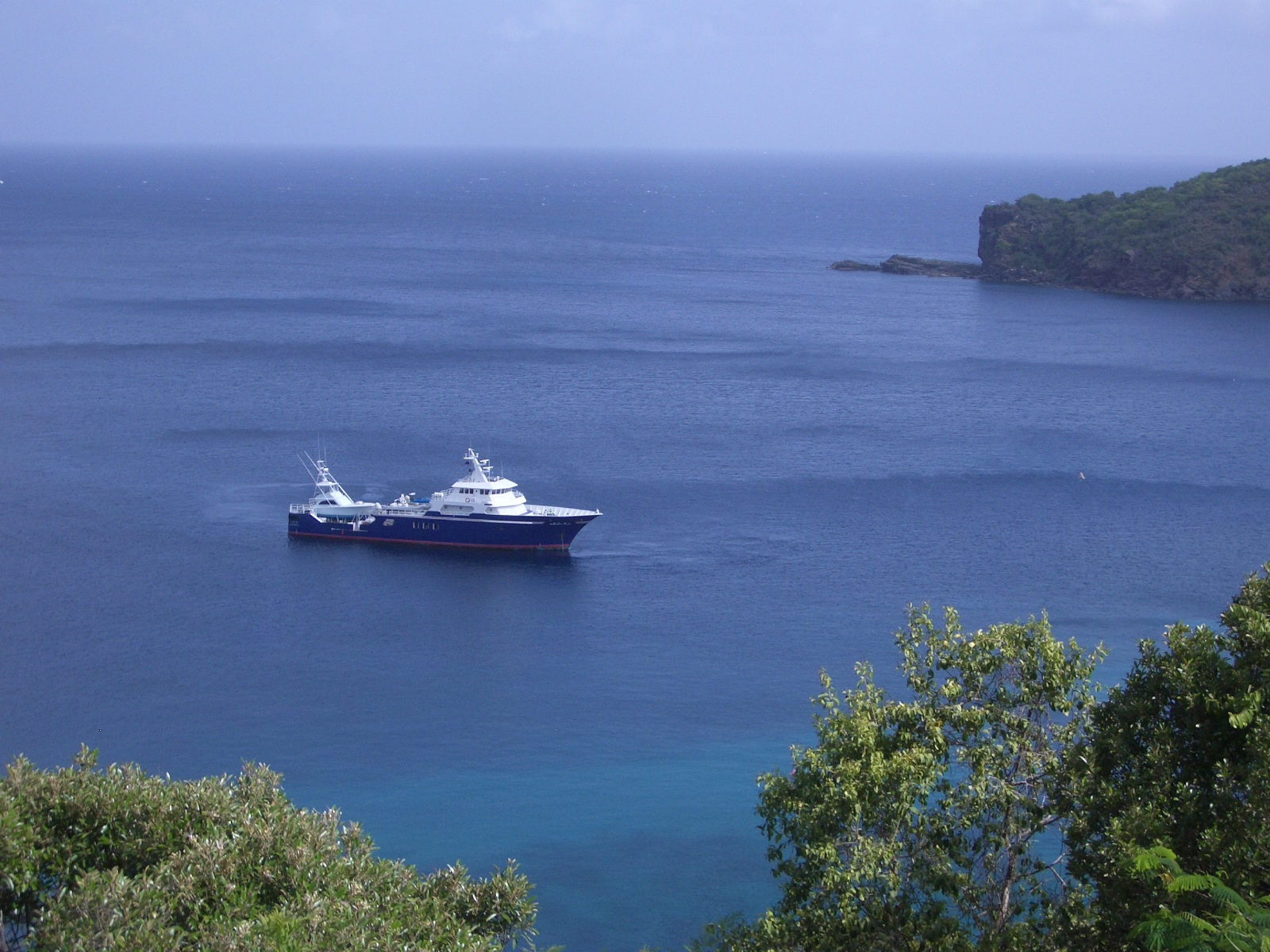

Supplement: S1 Fig — (TIF) [file pone.0144498.s001.tif]

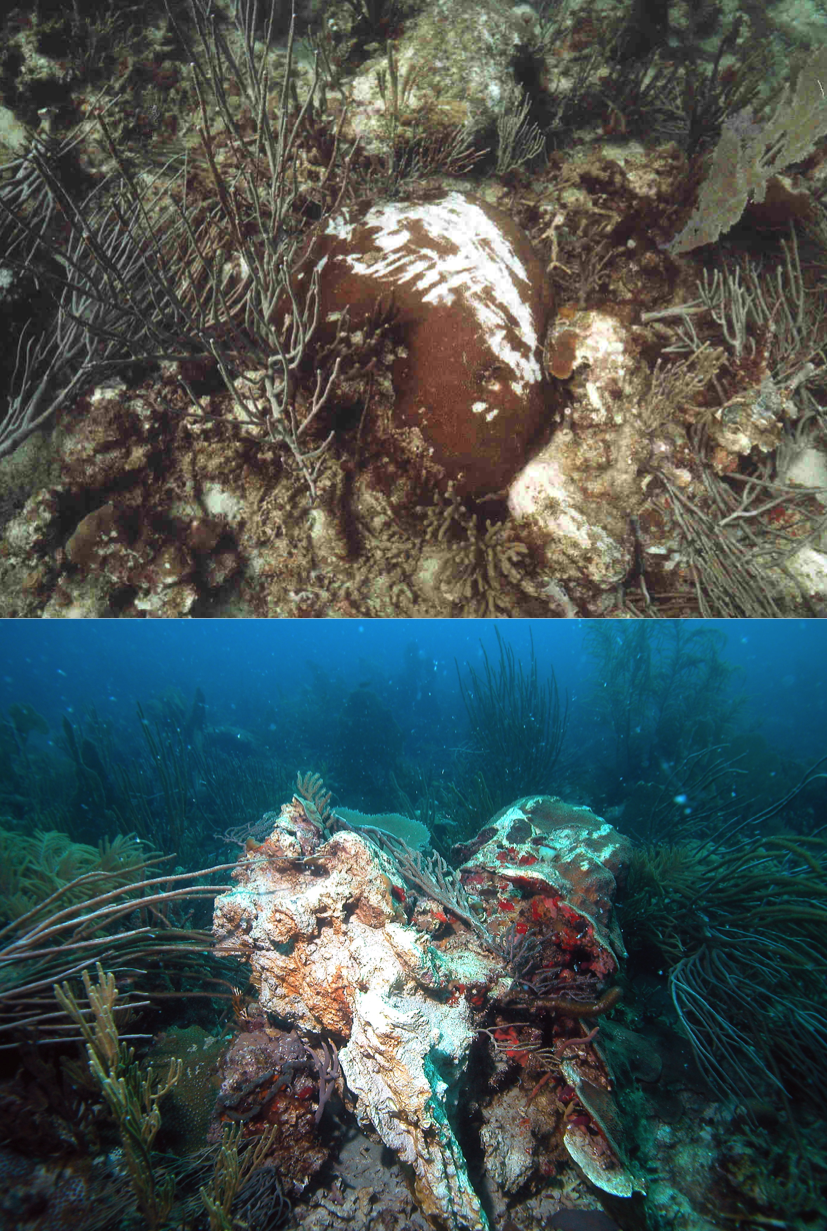

Supplement: S2 Fig — Photos taken in late July 2004. (TIF) [file pone.0144498.s002.tif]

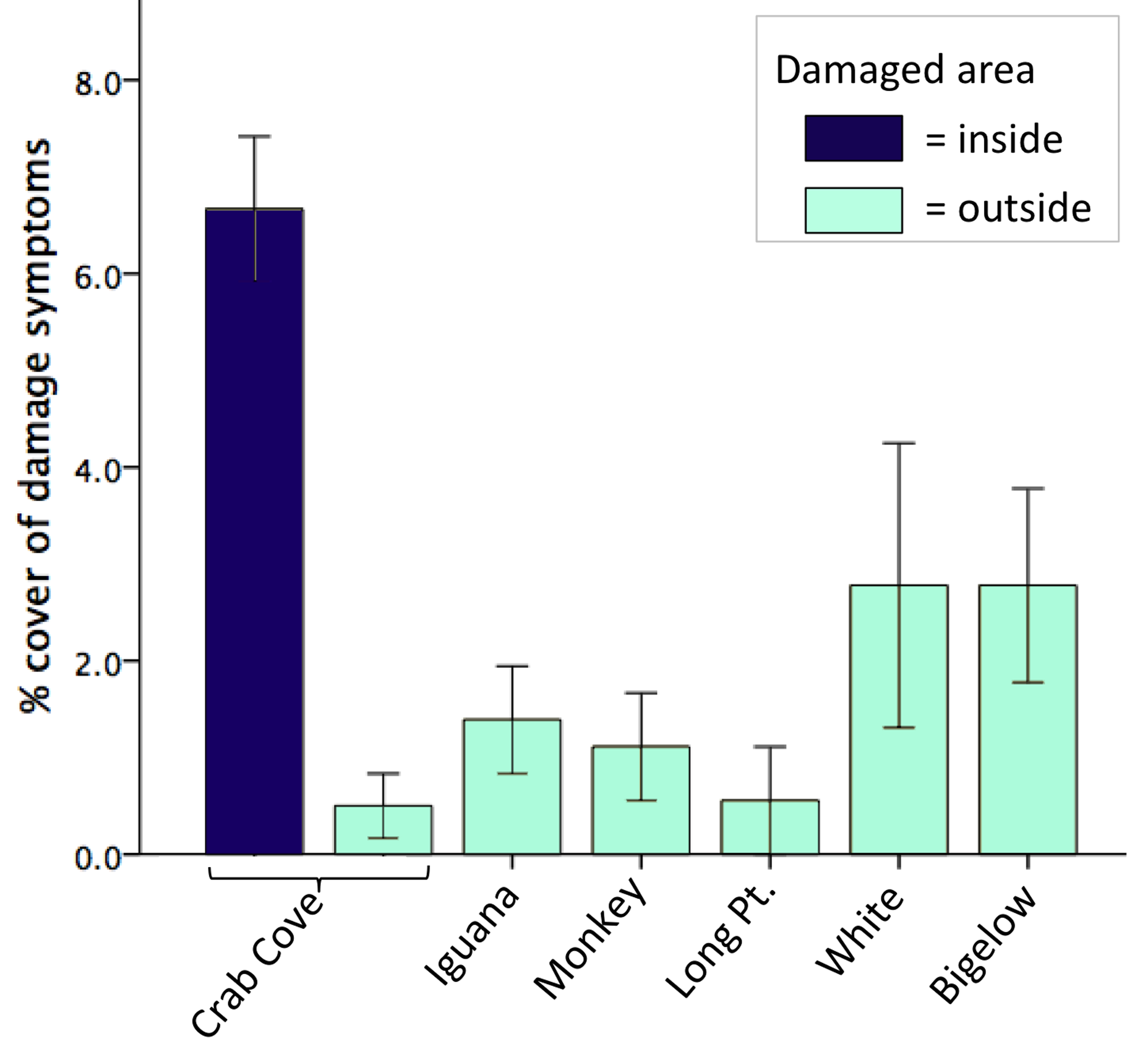

Supplement: S3 Fig — (TIF) [file pone.0144498.s003.tif]

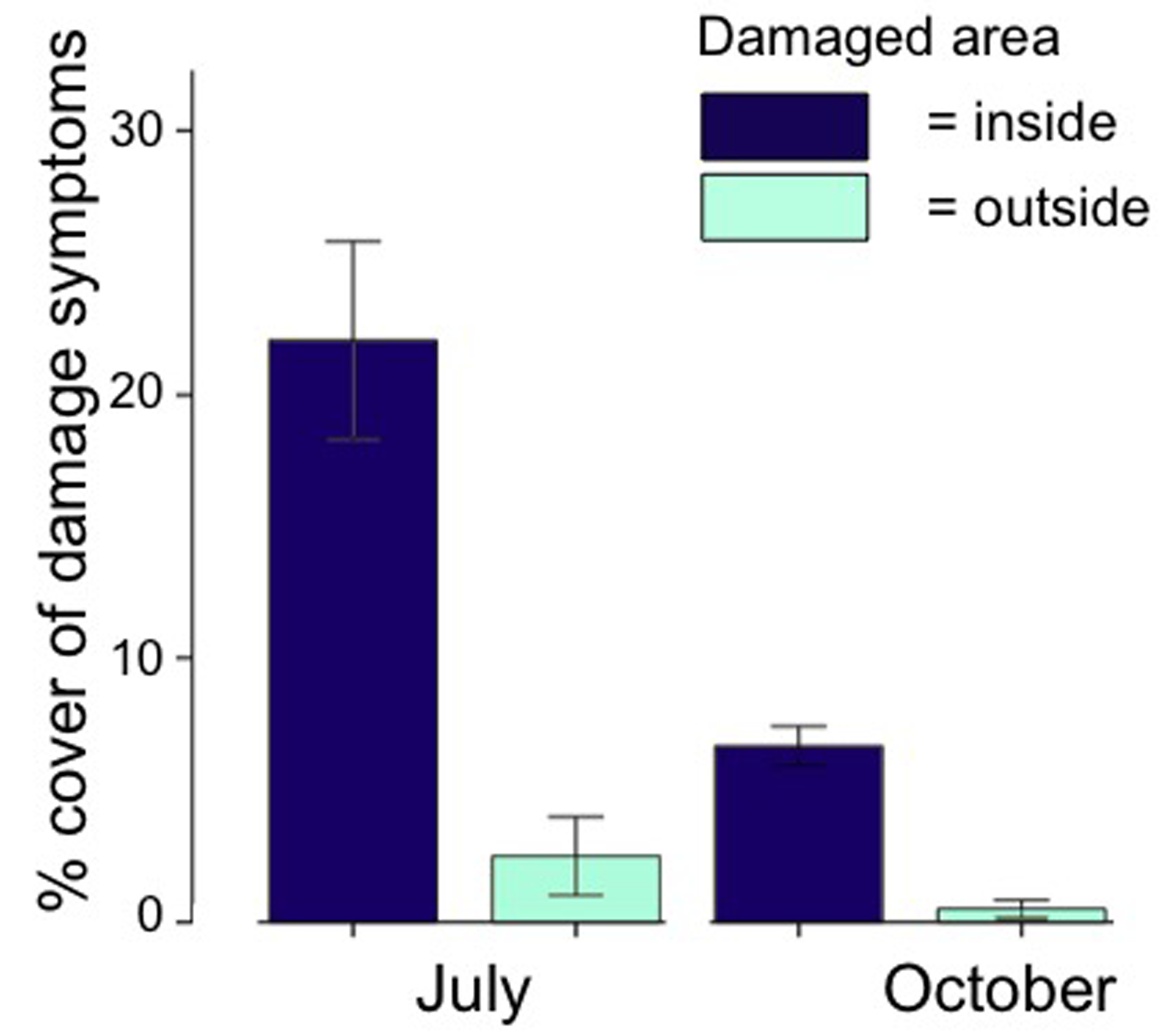

Supplement: S4 Fig — Damage symptoms in Crab Cove are shown alongside symptoms at 5 other sites around Guana Island. (TIF) [file pone.0144498.s004.tif]
