# Supplementary material for: Episodic Disturbance from Boat Anchoring Is a Major Contributor to, but Does Not Alter the Trajectory of, Long-Term Coral Reef Decline
Source: PLoS One. 2015 Dec 30;10(12):e0144498. doi: 10.1371/journal.pone.0144498 (PMC4696730; doi:10.1371/journal.pone.0144498)
Supplement: S1 Table — Sixty percent of fish counted belonged to these 17 species. (PDF) [file pone.0144498.s005.pdf]

S1 Table. A list of the most common fish species surveyed during the study

Family	Genus	Species
Acanthuridae	*Acanthurus*	*bahianus*
Acanthuridae	*Acanthurus*	*coeruleus*
Chaetodontidae	*Chaetodon*	*capistratus*
Haemulidae	*Haemulon*	*flavolineatum*
Labridae	*Halichoeres*	*bivittatus*
Labridae	*Halichoeres*	*garnoti*
Labridae	*Thalassoma*	*bifasciatum*
Pomacentridae	*Chromis*	*cyanea*
Pomacentridae	*Chromis*	*multilineata*
Pomacentridae	*Stegastes*	*leucostictus*
Pomacentridae	*Stegastes*	*partitus*
Pomacentridae	*Stegastes*	*planifrons*
Scaridae	*Scarus*	*iserti*
Scaridae	*Sparisoma*	*aurofrenatum*
Scaridae	*Sparisoma*	*viride*
Serranidae	*Hypoplectrus*	*puella*
Tetraodontidae	*Canthigaster*	*rostrata*
