# Supplementary material for: Episodic Disturbance from Boat Anchoring Is a Major Contributor to, but Does Not Alter the Trajectory of, Long-Term Coral Reef Decline
Source: PLoS One. 2015 Dec 30;10(12):e0144498. doi: 10.1371/journal.pone.0144498 (PMC4696730; doi:10.1371/journal.pone.0144498)
Supplement: S2 Table — (PDF) [file pone.0144498.s006.pdf]

S2 Table. Time-averaged mean percent coral cover (with SE) in the three zones within the Crab Cove site.

Zone	Mean cover (%)	SE
1	9.5	1.3
2	22.5	1.3
3	27.1	2.3
