# Supplementary material for: Episodic Disturbance from Boat Anchoring Is a Major Contributor to, but Does Not Alter the Trajectory of, Long-Term Coral Reef Decline
Source: PLoS One. 2015 Dec 30;10(12):e0144498. doi: 10.1371/journal.pone.0144498 (PMC4696730; doi:10.1371/journal.pone.0144498)
Supplement: S3 Table — (PDF) [file pone.0144498.s007.pdf]

S3 Table. Sample sizes for the linear mixed model. Table cells indicate the number of transects sampled within each zone in each year.

Anchor damage	Year	Zone		
		1	2	3
**Impacted**	1992	1	2	1
	1993		3	
	1994		1	
	1995		2	
	1996		1	1
	1997		2	2
	1998	2	2	
	1999		1	2
	2000		1	
	2001		2	
	2002	2	2	
	2003		1	3
	2004	1	12	5
	2005		7	
	2006	1	3	1
	2007		2	2
	2008		2	
	2009	1	2	1
	2010		3	
	2011		2	
	2012	1	3	1
	2013	1	2	
**Control**	1992		1	
	1993	2		1
	1994	2	1	
	1995			2
	1996		2	

1998			1
1999		1	
2000		3	
2001	1		1
2003	1	1	
2004	5	4	
2005		2	
2006	1		
2008		3	
2009	1		
2010		2	1
2011	2		1
2012	2		
2013			2
